# A constellation of mud volcanoes originated from a buried Arctic mega-slide, Southwestern Barents Sea

**DOI:** 10.1038/s41598-025-99578-5

**Published:** 2025-04-30

**Authors:** Claudio Argentino, Rune Mattingsdal, Tor Eidvin, Sverre Ekrene Ohm, Giuliana Panieri

**Affiliations:** 1 Department of Geosciences, UiT The Arctic University of Norway, 9037 Tromsø, Norway; 2 Norwegian Offshore Directorate, 9406 Harstad, Norway; 3 Retired from the Norwegian Offshore Directorate, 4003 Stavanger, Norway; 4 Department of Energy Resources, University of Stavanger, 4021 Stavanger, Norway; 5 Institute of Polar Sciences, National Research Council (CNR-ISP), 30172 Venice Mestre, Italy

**Keywords:** Geodynamics, Carbon cycle

## Abstract

Global estimates on the number of submarine mud volcanoes are highly uncertain, as well as their role in the deep-sea biosphere and methane budgets. Here, we report the discovery of ten Arctic mud volcanoes in the Barents Sea (440–480 m depth), where only two had been previously known. The new mud volcanoes form flat-topped mounds on the seafloor and are connected to seismic chimneys rooted within the infilling of a buried Pleistocene mega-slide scar. We suggest informally naming the area the Polaris Mud Volcano Complex. These structures have been active at least since the Late Weichselian deglaciation (< 20 ka), displaying evidence of ongoing methane-rich mud expulsion, i.e. mud pools and flows and chemosynthetic fauna. Finally, we propose a conceptual model for their formation which can be exported to other similar settings. Given the widespread occurrence of mega-slides and associated deposits along (paleo)glaciated continental margins, our findings call for a re-evaluation of mud volcanism potential in such regions.

## Introduction

Submarine mud volcanoes (MVs) are direct manifestations of Earth’s surface dynamics. They remobilize fluids (water, brine, hydrocarbons), sediment and breccia from the deep subsurface to the seafloor, providing a glimpse into buried structures and geological processes^1^. The key factors in the development of MVs are the presence of a deformable mud layer, and pressure gradients able to activate its movement. The latter are generated by the rapid accumulation of sediments and/or by lateral tectonic compression^2,3^ mainly developing at passive and convergent continental margins, respectively. The fluidized mud is squeezed from the source layer into faults or fractures and eventually expelled at the seafloor. Depending on the sediment viscosity and consolidation state, MVs form conical-shaped or flat-topped morphologies up to a few kilometers in size^2,4^. They are usually located in active petroleum basins suggesting a possible link between the presence of hydrocarbons and shale mobilization^5,6^. Overpressures generated by the diagenetic transformations of organic matter and buoyancy-driven factors could contribute to mud advection^7–9^. The gas contained in the expelled mud can escape the sediment via diffusion or as free gas bubbles potentially reaching the atmosphere. Methane emissions from submarine MVs contribute to greenhouse budgets^10,11^ for about 3.6 Tg per year^12^, corresponding to 1–2% of the global natural methane fluxes^13^ and to almost half of total oceanic fluxes ^14,15^. Yet, the estimated number of submarine MVs is highly uncertain^2^, ranging from a few thousand to several hundred thousand, thus affecting the accuracy of current estimates. This knowledge gap is mainly due to insufficient seafloor coverage with high-resolution mapping techniques and challenges in identifying and validating new offshore structures, which require ground-truth evidence acquired by means of underwater vehicles or sediment coring. This difficulty also increases the risk of geohazards to deep-sea operations in potential mud volcanic areas, i.e. constructions or drillings^3,4^. Submarine MVs also provide habitats and energy sources to the deep-sea biosphere. On the sediment surface, hydrocarbons contained in the mud fuel unique ecosystems relying on chemical energy instead of sunlight for carbon fixation^16,17^. Microbial mats typical of cold seep environments have been found in MVs worldwide^18^, and could serve as modern analogues for the Earth’s early biosphere with implications for the search for Life on other planets and moons^19,20^. Therefore, the research on MVs holds societal, economic, and scientific relevance.

The Arctic region hosts widespread offshore hydrocarbon resources^21^, and favorable geological conditions for upward sediment mobilization along the Eurasian and American continental margins, suggesting high potential for the development of MVs. However, despite thousands of cold seeps being mapped in the last decades in the Norwegian sector of the Barents Sea^22,23^, only two MVs were known from this area. The Håkon Mosby mud volcano (HMMV) was confirmed in 1995 on the southwestern Barents Sea slope at ~ 1260 m water depth^24^. It is a flat-topped MV standing ~ 15 m above the adjacent seafloor with a diameter of 1.4 km. Owing to its unusual position in a non-compressive setting and its association with shallow-subseafloor methane hydrates, it soon became one of the most studied systems globally^4,25,26^. In fact, of the known global MVs, only ~ 10% host gas hydrates^2^. It was only 28 years later that a new structure was discovered in the Barents Sea. In 2023, Remotely Operated Vehicle (ROV) investigations conducted during the AKMA3 expedition^27^ inside a seafloor crater located ~ 112 km to the north-east of HMMV, led to the discovery of an active gryphon (meter-scale cone-shaped edifice) emitting gas and mud. The whole area was named Borealis and is hypothesized to represent a collapse structure produced by past violent eruptive events^28^.

In this study we investigate ten seafloor mounds located just ~ 71 km north of Borealis, where openly-available 2D and 3D seismic data show a series of chaotic acoustic signals underneath. To ascertain the nature of these features and determine whether they are MVs, we conducted a dedicated ROV exploration and sampling campaign onboard R/V Kronprins Haakon during the EXTREME24 expedition in May 2024^29^. During the expedition we collected multibeam bathymetry and backscatter, seafloor imagery and sediment cores (eight gravity cores, one multicore and three push cores). Here, we report the results from geomorphological analyses, seismic interpretations, mud biostratigraphy and hydrocarbon geochemistry (oil and gas contained in the sediment), which provided clear evidence for an active MV system. Finally, we propose a conceptual model for the origin and evolution of these MVs which may be applicable to other areas on similar geological settings.

### Geological setting

The geological substrate underlying the investigated structures reflects the tectonic and depositional processes that followed the continental break-up of the Pangea and the opening of the Norwegian-Greenland Sea. Rifting started around the Paleocene/Eocene transition ~ 55 Ma and was followed by seafloor spreading that lasted until the Oligocene. Subsequent uplift and erosion of the Barents Sea continental shelf led to the deposition of a thick sediment wedge along the passive margin to the west. That wedge comprises pre-glacial marine deposits of Paleocene to Early Pliocene age (~ 55–2.7 Ma) overlain by Plio-Pleistocene glaciogenic sediments^30,31^. Those two units are separated by a major unconformity known as the Base Glacial (BG)^32^. The inception of large-scale but less frequent Pleistocene glaciations marked by repeated advances of the Scandinavian Ice Sheet to the shelf edge^31^ left a prominent erosion surface known as the Upper Regional Unconformity (URU). URU has an age ranging from 0.2 to 0.7 Myr ^31,33,34^ and extends over most of the Barents Sea. The sediments between BG and URU were deposited during smaller magnitude but more frequent Plio-Pleistocene glacial advances (pre-URU)^31^, which accumulated poorly compacted glaciogenic material on the continental slope via debris flow processes. Slope oversteepening and overpressures generated by high sediment inputs were critical preconditioning factors for extensive submarine landslides^32,35,36^ triggered by earthquakes^35^, gas hydrate destabilization^37^ or Late Pliocene volcanism^32^. More than 30 paleo-slides have been reported along the entire Norwegian-Barents Sea margin^35^, and recurring extreme mega-slide events (mobilized volume > 10^3^ km^3^) have been identified between 1.0 Ma and 0.2 Ma in our study area^32,35,36^.

## Results and discussion

### Geomorphological evidence of mud volcanism and cold seep habitat observations

**Fig. 1:**
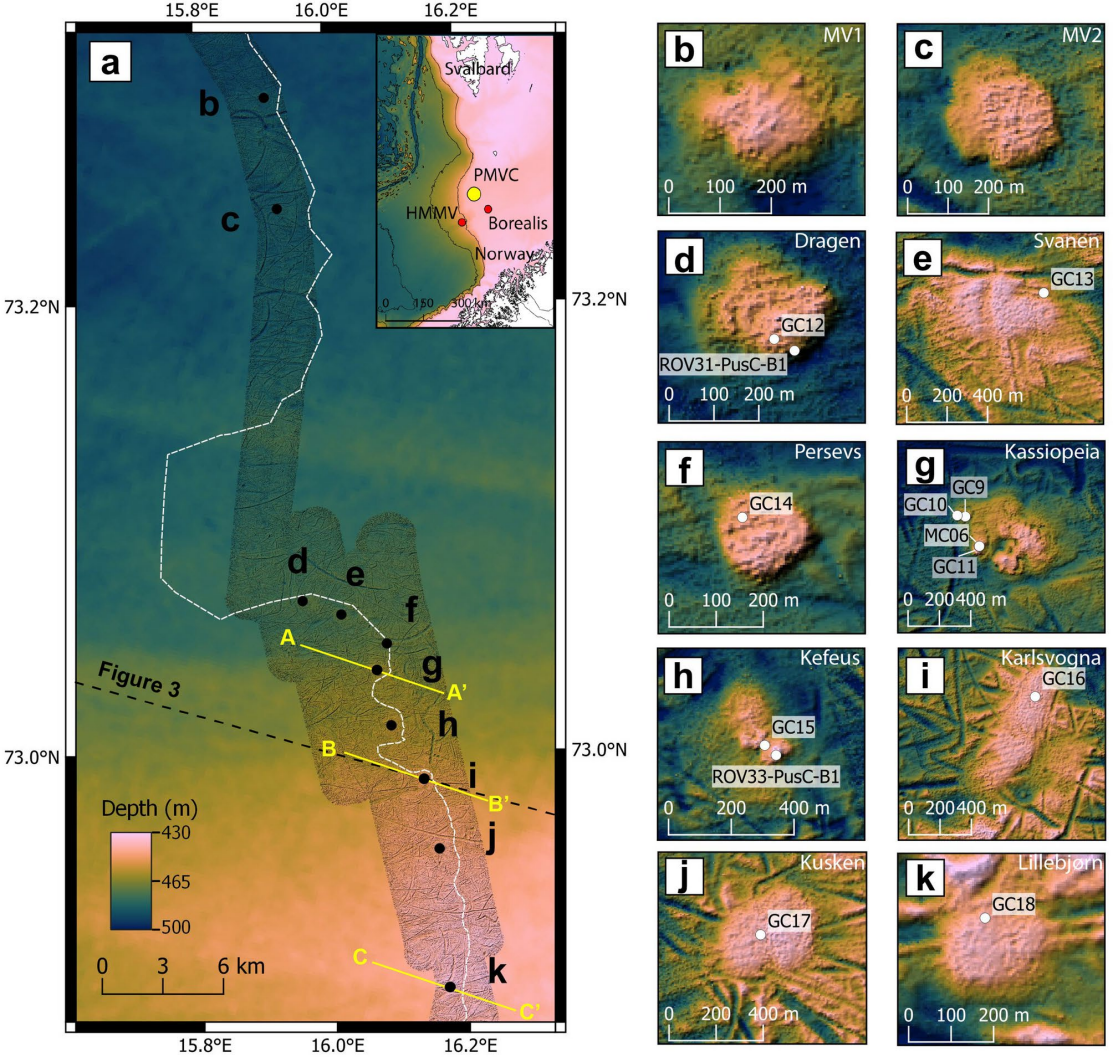
Overview of the study area showing the location and morphology of the Polaris Mud Volcano Complex (PMVC). (**a**) The inset map shows the geographic location of PMVC and the other two MVs in the SW Barents Sea, i.e. HMMV and Borealis. The area surveyed with high-resolution bathymetry is shaded, with IBCAO bathymetry in the background^45^. The ten mounds are indicated with black dots and are associated with letters from (**b**) to (**k**), referring to close-up views reported on the right. (**b**,**c**) have not been ground-truthed and are still unnamed. The color palette used in (**b–k**) has been adapted to emphasize the topography and does not match the depth scale in (**a**). The seafloor projection of the buried Pleistocene mega-slide head scar is shown with a dashed white line passing close to the mounds. The profile of the 2D seismic section of Fig. 3 is indicated with a NW–SE oriented dashed black line, whereas three shorter seismic profiles (AA′, BB′ and CC′) of Fig. 4 are shown in yellow. Color scale is accessible to people with color vision deficiencies^46^.

**Fig. 2:**
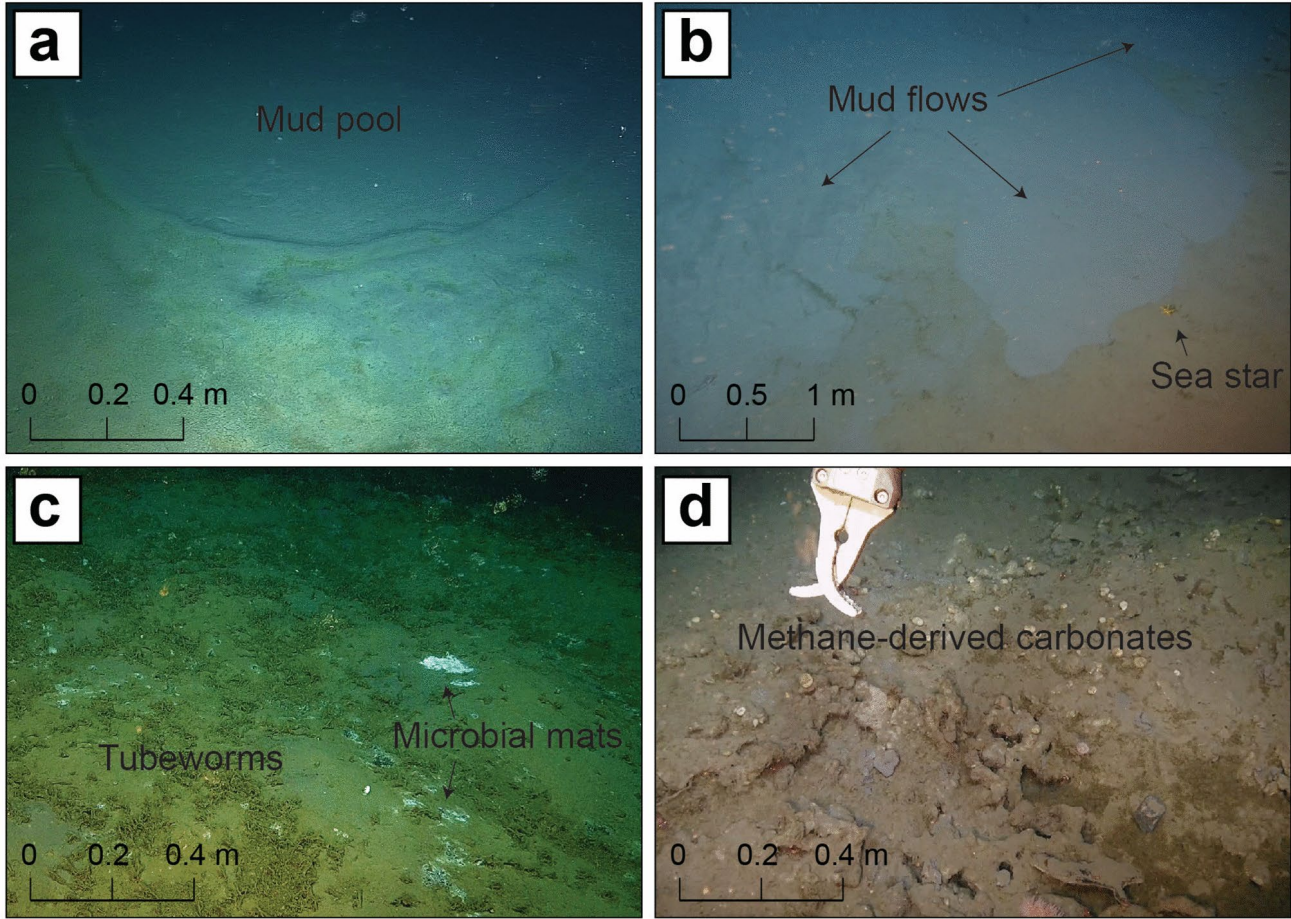
ROV observations of the seafloor on the mud volcanoes. (**a**) Mud pool with circular shape and diameter of less than 5 m found on Kassiopeia mud volcano. The inner side of the pool is composed of homogeneous fine-grained material of light-grey color and is separated from the surrounding brown sediment by a thin rim. The surface of the pool is flat and homogeneous without macrofauna. We identified four mud pools on Kassiopeia during the same dive. (**b**) Mud flow on top of the Kefeus mud volcano. The freshly emitted material is flowing over older deposits colonized by sessile and mobile fauna (i.e. sea star in the picture). (**c**) Chemosynthetic communities thriving on reducing fluids rising from the sediment at Kassiopeia. Tubeworm forests and white patches of microbial mats are scattered on the mound surface, indicating heterogeneous geochemical gradients underneath. (**d**) Methane-derived carbonates exposed on the seafloor at Karlsvogna.

We mapped ten mound structures over a 65 km-long composite bathymetric transect, spanning water depths from ~ 440 m to 480 m on the outer SW Barents Sea shelf (Fig. 1a). They are distributed along the NNW-SSE direction, with distances between 1.4 km and 19.5 km (Fig. 1a). All these structures have flat-topped morphologies and rounded (Fig. 1b–k) to ellipsoidal (Fig. 1i) shapes in plan view. The mounds have diameters and heights in the ranges ~ 200–900 m and 2–12 m, respectively (Table 1). The morphology of four of these structures (Fig. 1e,i,j,k) is smoother than in the surrounding background areas and on the other mounds (1 < topographic roughness index < 8). Two mounds (Fig. 1g,h) have particularly irregular surface topography, characterized by the presence of pits and features that suggest the merging of multiple structures. We obtained indirect lithological information of the material exposed at the seafloor from ship-based multibeam backscatter (Supplementary Fig. 1). The mounds display lower acoustic reflectance (low backscatter) compared to the background seafloor, suggesting a softer/homogeneous sediment texture, i.e. uncompacted mud. Two of the mounds exhibit single low backscatter flows originating approximately from their centers and developing toward the rim. In contrast, two other mounds show low-backscatter circular features, each with a diameter of less than 50 m, located either at the peaks of elevated sub-conical structures or within morphological depressions (Supplementary Figure S1). We conducted ROV video surveys on five mounds, which provided key geomorphological evidence of mud extrusion, including mud pools (Fig. 2a) and mud flows (Fig. 2b) (Supplementary Table 1, Supplementary Figure S1). Not all the backscatter anomalies were ground-truthed during the ROV dives. However, the elongated and circular low-backscatter features likely correspond to recent mud flows and small volcanic cones (gryphons)/collapse structures, similar to those commonly reported from submarine MVs worldwide^38–40^. No active gas bubbling was detected in the acoustic water column data nor observed during ROV dives. Still, the observation of widespread communities of tubeworms and microbial mats on five MVs (Fig. 2c) indicates an active cold seep environment supported by high fluxes of reduced compounds, i.e. H_2_S and CH_4_, toward the seafloor^39^. The presence of chemosynthetic habitats is associated with extensive methane-derived carbonate pavements (Fig. 2d; Supplementary Table 1), which suggests a protracted history of anaerobic methane oxidation. Bright spots in the backscatter map, matches carbonate outcrops observed at the seafloor (Supplementary Figure S1). Overall, our observations suggest that the MVs are currently in a quiescent state^7,40,41^, with localized and small-scale fluid expulsion activity.

**Table 1:** Summary of the geographical, morphological and compositional features of the mud volcanoes of the Polaris constellation.

Mud volcano	Latitude (DD)	Longitude (DD)	Avg. size w × d × h (m)	Mud biostratigraphy*	Total HC gas (ppm)	C_1_ %	C_2_ %	C_3_ %	δ^13^C-CH_4_ (‰ vPDB)	δD-CH_4_ (‰ vPDB)
MV1 (Fig. 1b)	73.2912	15.9120	300 × 300 × 5	N.A.	N.A.	N.A.	N.A.	N.A.	N.A.	N.A.
MV2 (Fig. 1c)	73.2418	15.9293	280 × 260 × 6	N.A.	N.A.	N.A.	N.A.	N.A.	N.A.	N.A.
Dragen (Fig. 1d)	73.0679	15.9581	310 × 280 × 12	Pleistocene	167,414	99.7	0.013	0	− 86.6	− 183
Svanen (Fig. 1e)	73.0617	16.0196	700 × 600 × 3	Pleistocene	188,480	99.5	0.007	0	− 75.4	− 198
Persevs (Fig. 1f)	73.0483	16.0866	200 × 176 × 6	Pleistocene	166,945	99.6	0	0	− 85.2	− 195
Kassiopeia (Fig. 1g)	73.0367	16.0662	700 × 530 × 6	Pleistocene	6,155	99.4	0.083	0	− 76.6	− 204
Kefeus (Fig. 1h)	73.0117	16.0920	450 × 400 × 4	Pleistocene	229,198	99.8	0.009	0	− 75.9	− 199
Karlsvogna (Fig. 1i)	72.9879	16.1402	900 × 550 × 2	Pleistocene	61,612	99.9	0.025	0	− 76.3	− 202
Kusken (Fig. 1j)	72.9567	16.1615	460 × 430 × 2	Pleistocene	213,081	99.7	0.012	0	− 76.5	N.A.
Lillebjørn (Fig. 1k)	72.8952	16.1742	330 × 270 × 6	Pleistocene	172,842	99.6	0.011	0	− 78.9	− 197

HC hydrocarbon, N.A. not analysed. *Mud biostratigraphy refers to the oldest age established based on foraminiferal assemblages.

Based on morphologies, we ascribe the investigated mounds to the same category of MVs known as mud-pies (flat topped mud volcanoes), produced by the extrusion of low-viscosity mud flowing easily across the seafloor^1,7,42^. Another example from this category is HMMV, which shows typical features of low-energy mud volcanism, i.e. flat and round-to-ellipsoidal morphology, generated by gradual pressure release rather than explosive processes. The MV-associated deposits either completely bury (Fig. 1i,j,k) of partially fill (northern part of MV in Fig. 1e) kilometer-long linear and curvilinear grooves that we interpret as ploughmarks incised by the keels of drifting icebergs during the Late Weichselian deglaciation^43,44^.

The new MVs are hereafter named after the brightest asterisms and constellations in the Arctic sky revolving around the North Star, using the Norwegian astronomical terminology, i.e. Dragen, Svanen, Persevs, Kassiopeia, Kefeus, Karlsvogna, Kusken and Lillebjørn. When referring to the entire MV complex, we recommend the informal use of the name Polaris, the North Star.

### A shallow fluid plumbing system rooted within the infilling of a buried Pleistocene mega-slide

**Fig. 3:**
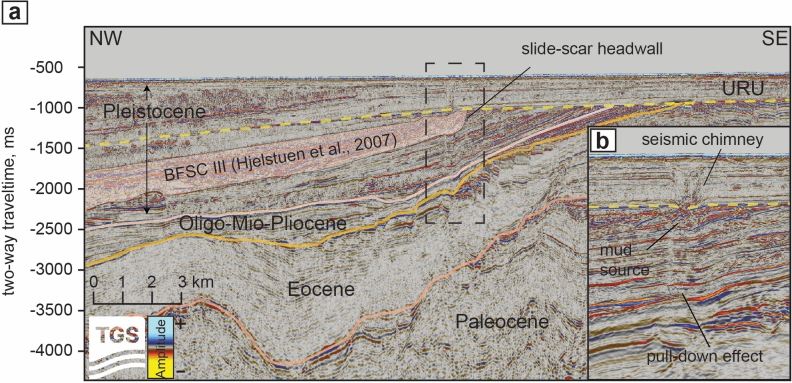
Interpreted NW- SE seismic section crossing a mud volcano in the study area. (**a**) We interpreted sedimentary successions ranging from Paleocene to recent, separated by two major unconformities marking the base of Plio-Pleistocene glacial sediments (pink color) and the erosive surface created by Pleistocene large-scale glaciations (URU; yellow dashed line). The sediment body remobilized during the mega-slide event is located further downslope and does not appear in the seismic section. (**b**) Close-up view of the area within the dashed box in image **a**, highlighting the seismic chimney and the pull-down anomalies beneath it.

We used publicly available seismic data from the Norwegian Offshore Directorate to explore the plumbing system underlying the mud volcanoes. In the dip-oriented seismic profile crossing our study site (Figs. 1a and 3), the base of the glacial units is located at around 2660 m (2500 ms) and is interrupted to the east by URU and its overlying flat-lying glacial units deposited during the Middle to Late Pleistocene. In the profile, URU is positioned at ~ 1800 m (~ 1500 ms) to the west and shoals up to ~ 1300 m (~ 1100 ms) to the east (Fig. 3). We identified a buried Pleistocene mega-slide as a highly heterogeneous unit within the seismic interval between 1,800 and 2,250 ms to the west of the profile and pinching out to the east against URU (Fig. 3). Internally, this unit comprises packages of continuous plane-parallel seismic reflectors with minor disturbances interbedded with totally deformed material. At the base of this interval, we identified the basal glide plane truncating the underlying reflectors and terminating to the east with a concave upward geometry representing the slide head scar. The seafloor projection of this feature, traced using 2D and 3D seismic datasets, coincides to the youngest generation of mass movements first visualized in 1993 in sequence stratigraphic analysis by Knutsen et al.^47^ and mapped in Kuvaas and Kristoffersen^32^. Following the chronostratigraphic frameworks for mega-slides in the SW Barents Sea presented by Hjelstuen et al.^35^, we attribute this unit to the Bjørnøya Fan Slide Complex III (BFSC III). This mega-slide event occurred between ~ 0.5 and 0.2 Ma and mobilized > 11,000 km^3^ of glaciogenic material that spread over an area of 66,000 km^2^.

**Fig. 4.**
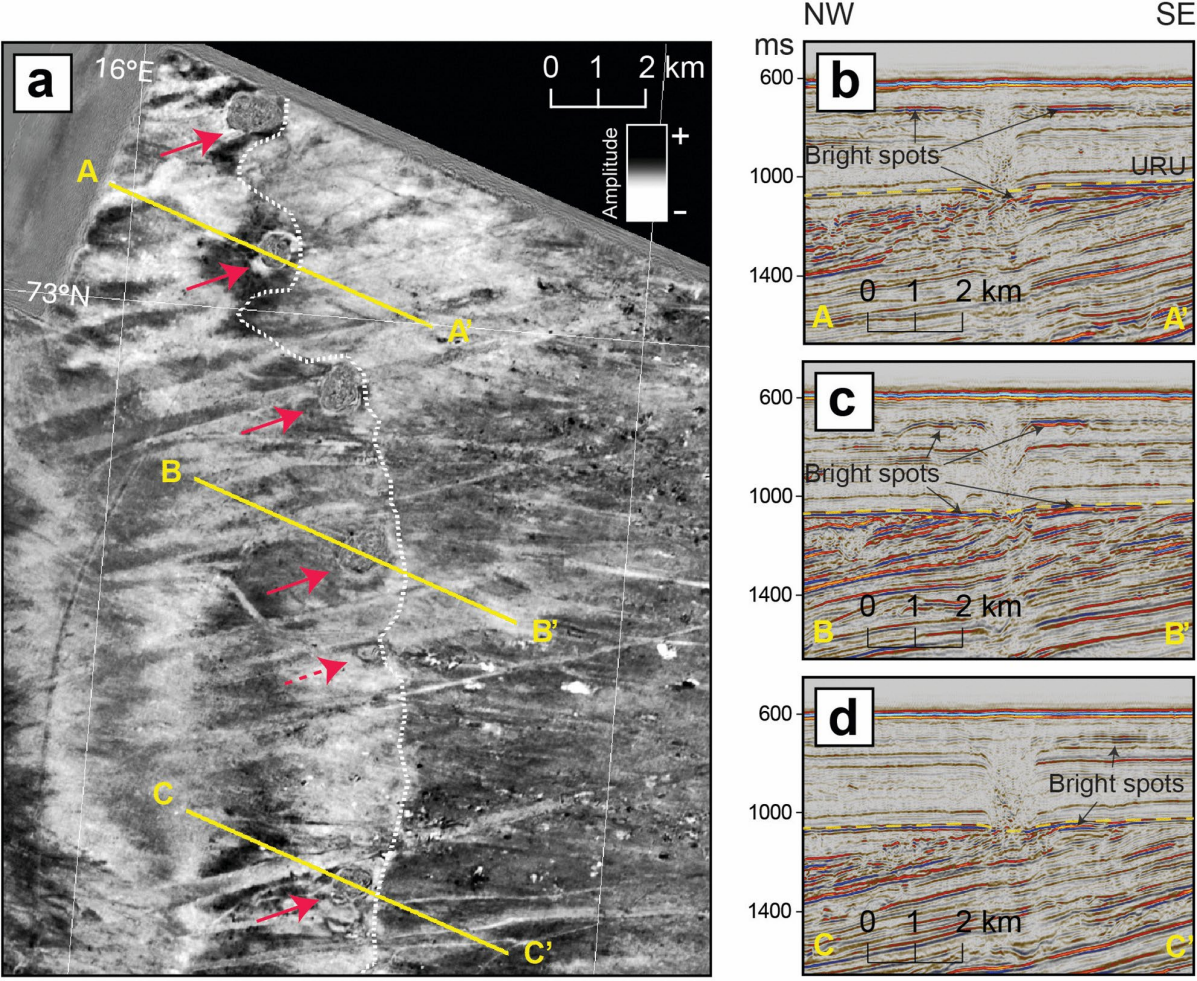
3D seismic data showing chimneys underlying the mud volcanoes. (**a**) Time slice at -844 ms two-way travel time showing amplitude, corresponding to approximately 200–250 m below seafloor, from 3D-seismic dataset SWB11. The seismic chimneys below five of the discovered MVs are indicated with continuous red arrows; one seismic chimney not linked to seafloor MVs is shown with a dashed red arrow. The dashed white line indicates the approximate location of the headwall of the buried slide-scar. (**b–d**) Seismic profiles A–A′, B-B′ and C–C′ from Inlines 2397, 2200 and 1944 of the 3D-seismic dataset SWB11. URU is observed at ~ 1050 ms. Negative amplitude anomalies appearing as bright spots indicate fluid accumulations beneath URU (yellow dashed line) and near the seismic chimneys.

The ten investigated mounds are distributed above the buried slide on the left/west side of the head scar projection (Figs. 1a and 3a). Each mound is underlined by a vertical zone of acoustic disturbance having semi-circular cross-section geometry (Figs. 3b and 4). Negative amplitude anomalies (opposed to seabed) observed at several stratigraphic levels in the adjacent strata suggests the presence of gas accumulations. Therefore, we interpret these vertical features as gas chimneys, which appear as seismically chaotic regions due to acoustic scattering caused by irregularly distributed, low-velocity gas-charged zones^48–50^. Our interpretation is consistent with the upward migration of gas-rich mud through vertical plumbing conduits, leading to seepage at the seafloor where mud volcanoes are formed. We also identified a chimney that does not reach the surface and disappears slightly above URU (Fig. 1a). As the chimneys represent fluidized mud rising through the sedimentary succession, the latter situation can correspond either to an extinct MV, whose activity terminated in the Late Pleistocene (with the top of the chimney corresponding to the paleo-seafloor), or to a nascent MV, with rising mud that has not yet reached the seafloor. This finding suggests that additional buried structures may exist north of the mapped slide area, where the sparse 2D seismic coverage hinders a spatial assessment of this process. Gas chimneys as the ones observed here are widespread in the southwestern Barents Sea, and correlate with structural elements separating the major basins and platforms^50,51^. Here, the gas chimneys originate below URU (Figs. 3 and 4a), but determining the precise location of the base of the chimneys is challenging due to the pull-down effect observed below the gas saturated zone. Pull-downs form under regions of reduced seismic velocities and cause apparent bending of the reflectors^48,50,52^. Thorough investigations of the 3D datasets allowed us to conclude that the gas chimneys are rooted in the sediments above the slide scar, and that the pull-downs crosscut that surface. This also explains why the seismic feature becomes less observable at increasing depth beneath the slide glide plane. Seismo-stratigraphic constraints imply that the sediment source for the MVs is composed of glaciogenic and contourite drift deposits which filled the space left at the slide scar after the mass waste event^32,35,53,54^. Benthic foraminiferal assemblages in expelled mud from the eight MVs correlate with Subzone NSB 16 × of King^55^ of Calabrian age (late early to late Pleistocene) (Supplementary Information), which is consistent with remobilization of ancient sediment from this interval. Mixed within these Pleistocene assemblages, we observed planktonic species typical of the Holocene and modern sediments, entrained on the way up to the seafloor and/or incorporated after deposition.

### Methane-rich sediments containing traces of oil

**Fig. 5:**
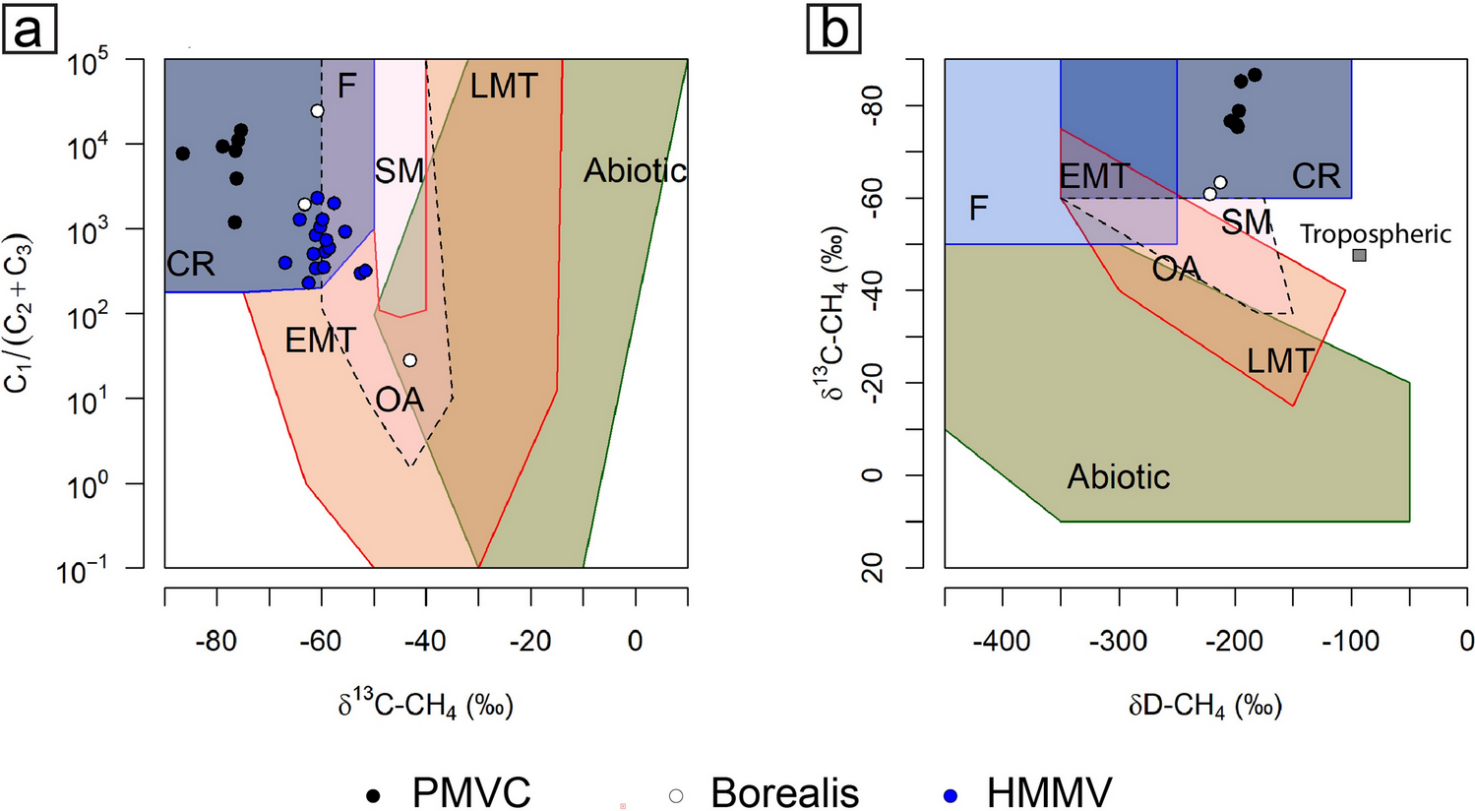
Geochemical composition of sediment-bound gas at PMVC (this study), Borealis^29^ and HMMV^57^. (**a**) Molecular ratio of methane (C_1_) over the sum of ethane (C_2_) and propane (C_3_) versus the stable carbon isotope composition (δ^13^C) of methane**.** All three MV areas fit in the range values of microbial gas^56^, although higher ethane and propane concentrations have been found in one sample at Borealis (**b**) Stable carbon and hydrogen (δD) isotope composition of methane. The hydrogen isotopic values allowed to attribute a carbonate reduction pathway for the methane being emitted with the mud at Polaris. Genetic fields (CR-CO_2_ reduction, F-methyl-type fermentation, EMT-early mature thermogenic gas, OA-oil-associated thermogenic gas, LMT-late mature thermogenic gas) after Milkov and Etiope^56^.

The systematic investigation of gas composition in gravity cores collected from the MVs (Fig. 1b–k) revealed high hydrocarbon contents, ranging from 6,155 (Persevs) to 229,198 ppm (Kefeus) (Table 1). Methane is the dominant hydrocarbon compound, ranging from 99.4 to 99.9% of the total hydrocarbon fraction. This molecular composition leads to an extremely dry gas with molecular ratios methane/(ethane + propane) from 1,192 to 14,385 (Fig. 5a), which is typically associated with microbial sources^56^. One sample collected from Svanen shows no traces of ethane and propane. The stable isotope composition (δ^13^C, δD) of methane shows consistent values ranging in δ^13^C between − 86.6 to − 75.4 ‰ and in δD values between − 204 to − 183 ‰. These values cluster in the genetic field of the carbonate reduction pathway, indicating a microbial gas source for the methane (Fig. 5b).

The sediment contains low amounts of extractable organic matter, ranging from 78 to 132 mg/kg, of which saturate and aromatic hydrocarbons represent less than 20% and 32%, respectively. The n-alkane distribution in the gas chromatograms of the samples displays an evident hump (Supplementary Figure S2) generated by the presence of an unresolved complex mixture indicating biodegradation. The preference of odd-numbered components in the n-C_23_ to n-C_33_ range further suggest them to be derived from an immature/early mature source, also consistent with hopane composition (Supplementary Figure S3). This source has been deposited in an open to shallow marine/coastal environment, according to the sterane-based proxies (Supplementary Figure S4) and Pristane (Pr)/n-C_17_ and Phytane (Ph)/n-C_18_ ratios (Supplementary Figure S5). The relatively high concentration of iso-alkanes relative to n-alkanes corroborates ongoing biodegradation (Supplementary Figure S2).

### Origin and evolution of the Polaris mud volcanoes

The plumbing system of PMVC, which extends only about 300–350 m into the sedimentary column, stands out as an unusual feature in the context of submarine MVs. Many submarine MVs are associated with deep-rooted systems^4,6^, often reaching up to several kilometers below the seafloor, e.g. Calabrian arc (central Mediterranean Sea)^40^, central Black Sea^58^, Gulf of Cadiz^59^, Taiwan accretionary prism^60^. Polaris MVs are somewhat similar to the shallow-rooted systems reported from Lake Baikal^61^, Russia. In Lake Baikal, the upward migration of warm fluids through faults and fractures causes local gas hydrate destabilization, leading to sediment remobilization and the formation of MVs. Differently from the Lake Baikal examples, there is no bottom-simulating reflector (seismic feature indicating the base of the hydrate stability zone) underneath the Polaris MVs. Our thermodynamic calculations considering pure methane hydrate stability at PMVC indicate that the edge of the hydrate stability zone (where the bottom of the gas hydrate stability zone intersects the seafloor) pinches out along the 430 m isobath. The stability zone penetrates ~ 60 m in the deepest and northernmost volcano site (MV1) and only a few meters in the shallowest and southernmost one (Lillebjørn). Despite their potential stability, we did not retrieve any gas hydrates during sediment coring nor observed “soupy sediments” ^39,62,63^ or degassing structures indicative of ex-situ hydrate destabilization. In a previous expedition, gas hydrates were successfully collected from the nearby HMMV (~ 1200 m depth) using the same non-pressurized gravity corer used in the present study^17^. Since Polaris is located at much shallower depths than HMMV, we rule out the possibility that complete hydrate dissociation happened during core retrieval and onboard activities, so we conclude that shallow hydrates are absent in the study area. This is further supported by seafloor reflectivity data showing that the recent mud flows on Dragen and Persevs are associated with low backscatter (Supplementary Figure S1), which is another indication of degassed, hydrate-free sediment^39^. Local positive temperature anomalies associated with MVs^28,39,64,65^ can reduce the theoretical thickness of the hydrate occurrence zone by up to one hundred meters^61^, thus shifting the MVs in the Polaris area out of the stability zone. In addition, brine advection could also contribute to reduce the stability zone^66^, but there are no observations of brine pools from our ROV dives. We did not conduct CTD casts or heat probe surveys on top of the MVs, which prevents us from fully assessing these hypotheses. We cannot rule out the possibility of paleo-gas hydrate occurrence in this area, based on available data. Nonetheless, our datasets and the geological setting suggest different mechanisms to explain the inception of shallow-rooted mud volcanism at Polaris, rather than the shallow gas hydrate destabilization scenario proposed for the Lake Baikal MVs^61^.

**Fig. 6:**
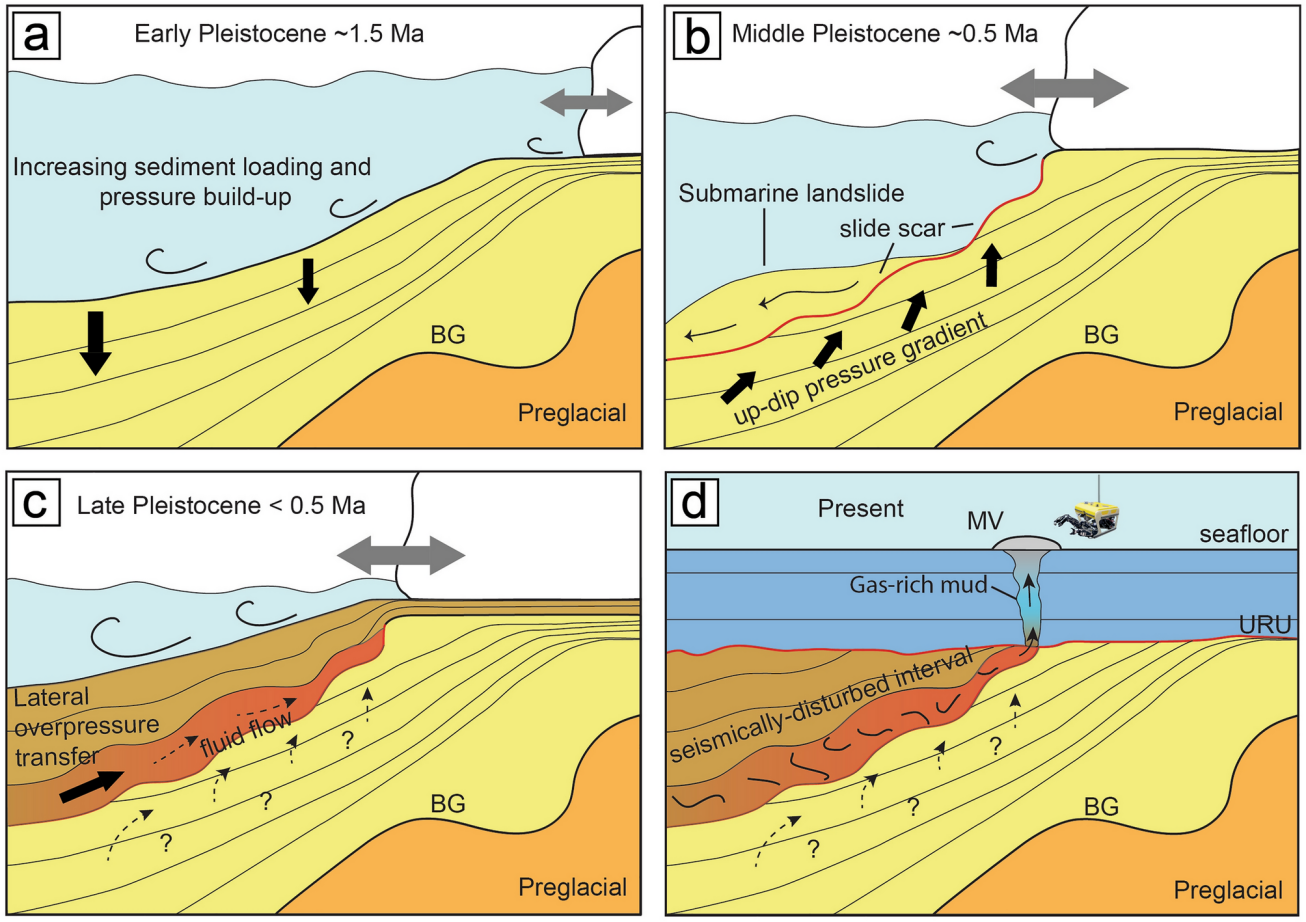
Conceptual model for the formation of PMVC. (**a**) Deposition of glaciogenic sediment on the western Barents Sea paleo-slope started in the Late Pliocene and continued in the Pleistocene. Enhanced lateral transport of material from the shelf resulted in pressure build-up and slope steepening, creating favorable conditions to mass failure. (**b**) With the intensification of glaciations after ~ 1 Ma, the sediment load on the slope further increased and led to recurrent mega-slide events along the continental margin (here only the one associated with mud volcanism is shown for simplicity), likely triggered by earthquakes^35^. As a result, a pressure gradient generated in the slide scar area. (**c**) The slide event left a zone of weakness along the scar surface, filled by continuous glaciogenic sedimentation and contourite drift sediment which induced disequilibrium compaction and increased pore pressure. Overpressures were transferred laterally from deeper to shallower areas, following the stratigraphy. (**d**) The accumulation of overpressures in the head scar region eventually resulted in near-lithostatic pressure, which was necessary to initiate the upward migration of gas-rich mud. What we observe today from seismics is fluidized mud migrating along the basal glide plane and rising in proximity of the seafloor projection of the head scar. The sediment package overlying the slide plane is seismically disturbed. MV = mud volcano; BG = base glacial (see main text).

We propose a conceptual model for the inception of mud volcanism at PMVC, which is linked to modifications of the overburden stress field on the slide scar. Our model fits in the frame of the Northern Hemisphere Glaciations (~ 2.7 Ma in the Late Pliocene), when the uplift and erosion of the Barents Sea shelf drastically shaped the western margin (Fig. 6a). The repeated short-term expansion of the NW European ice sheet eroded fine-grained material from sub-cropping Cretaceous and Paleogene successions on the Barents Sea shelf to form large fans on the adjacent slope (Fig. 6a). This process is documented by 2D seismics and drillings along the entire western margin^53,67,68^. The rapid sediment deposition progressively loaded the soft Oligo-Mio-Early Pliocene pre-glaciogenic substrate made of hemipelagic oozes^35^ and overlying contourite drift sediment^53^ intermingled with the Late Pliocene–Pleistocene glaciogenic sediments. Previous studies^31,32,35^ indicated several mega-slide events occurring after the onset of frequent shelf-edge glaciations, which started at ~ 1 Ma^31^. Also, the mega-slide identified in our study formed at that time. In that period, enhanced lateral transport caused further oversteepening and overpressure accumulation within slope material, creating favorable conditions for recurrent mass failures, which were most likely triggered by small to medium-scale earthquakes^69^ (Fig. 6b). Failure surfaces typically occurred within contouritic sediments having higher clay and water contents, plasticity and liquidity index compared to glaciogenic debris-flow packages^70,71^. Numerical simulations^72^ have shown that rapid sediment loading of glaciogenic deposits on high-latitude margins can generate overpressures in fine-grained contourites. The displacement of the slide mass, which is now located further downslope than the area shown in the seismic section in Fig. 3, caused a sudden decrease in overburden in the slide scar and an increase in sediment load at its foot. As a result, along-dip pressure gradients generated in the slide scar area. A similar scenario was described for the initial stage of HMMV, as a response to the Bear Island Slide event^73^. In the Polaris area, the rapid filling of the slide scar with new debris flows and fine-grained contouritic deposits induced disequilibrium compaction and increased pore pressure (Fig. 6c). Disequilibrium compaction is indeed the most common mechanism of overpressure generation within rapidly-deposited muddy sediments^74^. In-situ methanogenesis or hydrocarbon migration from underlying successions might have partially contributed to pore fluid overpressure^7^, although those conditions are commonly associated with deep-rooted MVs^1,6^. Owing to the relatively reduced sedimentary column, sediment loading was not enough to reach near-lithostatic overpressure levels required for inducing piercement of overlying strata. 3D seismic data (Fig. 3) suggests that the direction of fluid movement (NW–SE) roughly follows the sub-horizontal stratigraphy of enclosing glaciogenic successions. This continues until the fluids reach the slide wall to the east, from which vertical gas chimneys depart (Figs. 3 and 4). We propose that the inclined geometry of the scar surface and overlying deposits facilitated the lateral transfer of overpressures^75–77^, which accumulated toward the head zone. The slide scar acts as a highly fractured weak zone that allows fluids to move through^73,78^, facilitating overpressure dissipation and hydrofracturing of the overlying strata. This process would have enabled the vertical migration of fluidized, gas-rich mud toward the seafloor (Fig. 6d). Based on the available data, we cannot completely rule out the possibility that gas hydrates existed prior to the mega-slide event, and their destabilization following the mass waste may have concurrently contributed to triggering fluid mobilization.

The timing for the inception of sediment remobilization and volcanism remains uncertain, but the high-resolution bathymetry^79^ shows that the mounds cover the iceberg ploughmarks, i.e. (sub)linear erosional features carved by the icebergs as they crossed this area during the Late Weichselian deglaciation (< 20 ka)^43,80,81^. Therefore, the MVs deposits currently observed must have formed later, as they show no signs of glacial erosion, i.e. iceberg scouring. A similar interpretation was also made for Borealis^29^. Differently from HMMV, where the identification of buried paleo-craters allowed the reconstruction of ~ 300 ky-long MV activity with phases of reactivation^73^, here we do not observe buried paleo-features. The gravity cores and carbonate samples collected from the mounds will be target of future sedimentological and geochemical analyses and U/Th dating, respectively, helping us to reconstruct the dynamic and history of methane fluxes and mud volcanism. Borealis is indeed different from HMMV and Polaris both in terms of its geological features and its formation mechanism. Borealis is characterized by four large seafloor depressions hosting widespread carbonate outcrops and intense methane seepage. The formation mechanism for Borealis MVs proposed by Panieri et al.^29^ suggests that their origin is tied to the accumulation of overpressure beneath the surface, which eventually dissipated through explosive events. These explosive events are thought to be caused by the destabilization of gas hydrates due bottom water warming and pressure drop associated with the last deglaciation*.* That process was enhanced by upwelling warm fluids, which acted as a secondary trigger by promoting further destabilization of gas hydrates. The flat-topped geomorphologies of Polaris mounds and HMMV suggest that recent mud extrusion has been associated with gradual pressure release rather than explosive processes^7,39^. The relatively low hydrostatic pressure acting on the shallow plumbing system of Polaris, coupled with the absence of hydrate deposits in the subsurface, makes the possibility of future catastrophic eruptive events unlikely. Still, the rearrangement in the distribution of mud extrusion features (pools, flows) and cold seep habitats on the MVs might be expected on temporal scales ranging from a few years to a few thousand years^82,83^.

Owing to the widespread occurrence of mega-slide deposits along (paleo)glaciated continental margins, the discovery of a constellation of MVs in the Polaris area calls for a re-evaluation of the mud volcanism potential in these regions with geodynamic implications. Since the number of known submarine MVs worldwide is highly uncertain^2^, these areas should become important targets for future exploration aiming at reducing this gap in knowledge. Moreover, joint multidisciplinary efforts are necessary to better understand their contribution to the deep-sea biosphere and the methane cycle.

## Methods

### Hydroacoustic surveys and seismic databases

During the EXTREME24 expedition^29^, we used a EM710 multibeam echosounder (MBES) running at 70–100 kHz range for acquiring high-resolution bathymetry and acoustic backscatter data. The system is mounted on the port drop keel of the ship and is particularly suited for swath bathymetry surveys up to 800 m water depth. The system sends out 400 beams at an angle of up to 700 on each side. We analyzed the water-column data using QPS FMMidwater to identify gas bubbles rising from the seafloor. We used QPS Fledermaus DMagic to grid seafloor bathymetry, and Fledermaus FMGT to process the backscatter data. The topographic roughness index used to estimate the degree of irregularity of the seafloor at the different volcanoes was calculated in QGis by the largest inter-cell difference of a central pixel and its surrounding cell as defined in Wilson et al.^84^. The seismic data that we used in this study is openly available in Diskos (https://www.diskos.com), the Norwegian national repository of seismic, well, and production data, and from some multiclient proprietary data. The 2D-seismic line used in Fig. 3 is a multiclient proprietary CFI 2D-line from TGS. The 3D-seismic used in Fig. 4 is from public 3D-dataset SWB11, a 5 313 km^2^ seismic cube acquired in 2011.

### ROV imagery

We acquired seafloor imagery using a SUPPORTER 32-type ROV owned and operated by REV Ocean. This ROV is equipped with two forward-facing Orca HD (IP) cameras and a separate SubC Rayfin 4 K camera with a strobe, all mounted on pan-and-tilt assemblies. Additional cameras were installed for operator situational awareness, piloting, and sampling operations. The vehicle’s forward-facing lighting capacity includes sixteen LED lights with a combined output of 120,000 lumens.

### Sediment coring and gas sampling

We conducted gravity coring in eight MV locations using a 6-m long corer lowered from the back of the vessel using a A-winch. The sediment was collected into plastic liners introduced into the barrel prior to sampling operations and kept in position with a core cutter. A core catcher placed between the bottom of the liner and the cutter prevented sediment loss upon retrieval. Back on deck, we sampled the material trapped in the core catcher for biostratigraphic analyses and sediment-bound oil geochemistry. The latter was wrapped in aluminum foil and kept frozen at − 20 °C. We sampled sediment-bound gas by placing a ~ 200 mL slice of sediment taken with a spatula from the bottom of the core into an IsoJar™ paintcan container (Isotech Laboratories and Humble Instruments, USA), to which we added 0.5 mL of 10% benzalkonium chloride as antimicrobial agent and tap water. Gas samples were stored upside-down at 4 °C until onshore analyses were performed within one month.

### Biostratigraphy

Sediment samples for biostratigraphic analysis were collected from the core catchers of gravity cores GC9, GC10, GC11, GC12, GC13, GC14, GC15, GC17, GC18, from the bottom of multicore tube MC-06_006 and from push core ROV31-PusC-B1, for a total of 11 samples. The undried, unconsolidated samples were soaked in water, wet sieved and dried. Then the dry sample material was split into fractions. The fractions of 0.1–0.5 mm were gravity-separated in heavy liquid. The air in the foraminiferal chambers caused them to float up, and the tests could be collected for identification. The fraction less than 0.06 mm (silt and clay) were washed down the sink. The other fractions were used for simplified grain distribution analyses. The fractions larger than 0.5 mm and less than 0.1 mm were also examined to investigate whether any important foraminifera were left in these fractions. All foraminifera and some other microfossil of importance from all fractions were recorded (more than 300 specimens in each sample).

### Hydrocarbon geochemistry

Headspace gas analyses were conducted at the laboratories of Applied Petroleum Technology (APT) in Oslo, Norway, on 8 sediment samples collected from gravity cores GC11, GC12, GC13, GC14, GC15, GC16, GC17, GC18 (Fig. 1d–k; Table 1). Aliquots of gas for molecular analyses were injected into an Agilent 7890 RGA GC equipped with Molsieve and Poraplot Q columns and measured on a flame ionisation detector (FID). The carbon and hydrogen isotopic composition of methane was determined on a Trace 1310 gas chromatograph (Thermo Fisher Scientific), equipped with a Poraplot Q column and PTV (Programable Temperature Vaporizing) injector. The GC was interfaced via GC-Isolink II and Conflo IV to a Delta V Isotope Ratio Mass Spectrometer (IRMS; Thermo Fisher Scientific). Precision on δ^13^C and δ^2^H was better than 1 ‰ vPDB and 10 ‰ vSMOW, respectively. Oil preparation and analysis followed NIGOGA guidelines (Norwegian Industry Guide to Organic Geochemical Analysis, 4th Edition), and were also conducted at APT in Oslo, on 8 samples from GC11, GC12, GC13, GC14, GC15, GC16, GC17, GC18 (Fig. 1d–k). For the extractions, each sample was weighed accurately into glass vials with approximately 80 cc of dichloromethane with 7% (vol/vol) methanol and placed in ultra-sound bath for 1 h. An aliquot of 10% of the extract was transferred to a pre-weighed bottle and evaporated to dryness. For stable carbon isotope analyses of the hydrocarbon fractions, the samples were dissolved in dichloromethane and 20 µL was transferred to a tin capsule. The solvent was evaporated in an oven at 50 °C. The samples were then loaded into an automatic sampler which then placed them into a combustion reactor (Thermo Fisher Scientific Elemental Analyzer) held at 1020 °C. CO_2_ is separated by column and flashed into Delta V Plus Isotope Ratio Mass Spectrometer (IRMS) (Thermo Fisher Scientific) via Conflo IV. Gas chromatographic analyses of extractable organic matter were conducted on a HP Agilent 7890A GC Gas Chromatograph using a CP-Sil-5 CB-MS column, length 30 m, i.d. 0.25 mm, film thickness 0.25 µm. Analyses of saturated and aromatic hydrocarbons were conducted via GC–MS using a Thermo Scientific DFS™ magnetic sector mass spectrometer. The instrument was tuned to a resolution of 3000 and data were acquired in Selected Ion Recording (SIR) mode. The column used was a 60 m CP-Sil-5 CB-MS with an i.d. of 0.25 mm and a film thickness of 0.25 µm.

### Modelling of gas hydrate stability

The modern gas hydrate stability zone was calculated for the depth range 440–480 m by assuming steady state conditions and applying reference values of bottom water temperature of 4 °C, salinity of 35 PSU from background areas^28^, and the average geothermal gradient for the SW Barents Sea of 36 °C/km^85^. We did not collect CTD data from the investigated mounds, which hinders more accurate thermodynamic modeling. The model was implemented using the graphical user interface CAGEHYD^86^ based on the CSMHYD code^87^. The model was run for pure methane hydrates, following the gas composition of headspace samples measured in this study.

## Supplementary Information

Supplementary Information.

## Data Availability

Data is provided within the manuscript or supplementary information files. High-resolution bathymetry of the PMVC is openly available in Dataverse.NO at https://doi.org/10.18710/TCARF3.
