# Author Correction: A constellation of mud volcanoes originated from a buried Arctic mega-slide, Southwestern Barents Sea

**DOI:** 10.1038/s41598-025-07150-y

**Published:** 2025-06-27

**Authors:** Claudio Argentino, Rune Mattingsdal, Tor Eidvin, Sverre Ekrene Ohm, Giuliana Panieri

**Affiliations:** 1 Department of Geosciences, UiT The Arctic University of Norway, 9037 Tromsø, Norway; 2 Norwegian Offshore Directorate, 9406 Harstad, Norway; 3 Retired from the Norwegian Offshore Directorate, 4003 Stavanger, Norway; 4 Department of Energy Resources, University of Stavanger, 4021 Stavanger, Norway; 5 Institute of Polar Sciences, National Research Council (CNR-ISP), 30172 Venice Mestre, Italy

Correction to: *Scientific Reports* 10.1038/s41598-025-99578-5, published online 30 April 2025

The original version of this Article contained an error in Figure 1, panel g, where the southernmost gravity core “GC11” was incorrectly given as “GC10”. In addition, in panels b and c, names for the mud volcanoes were omitted. The original Fig. 1 and accompanying legend appear below.

**Fig. 1:**
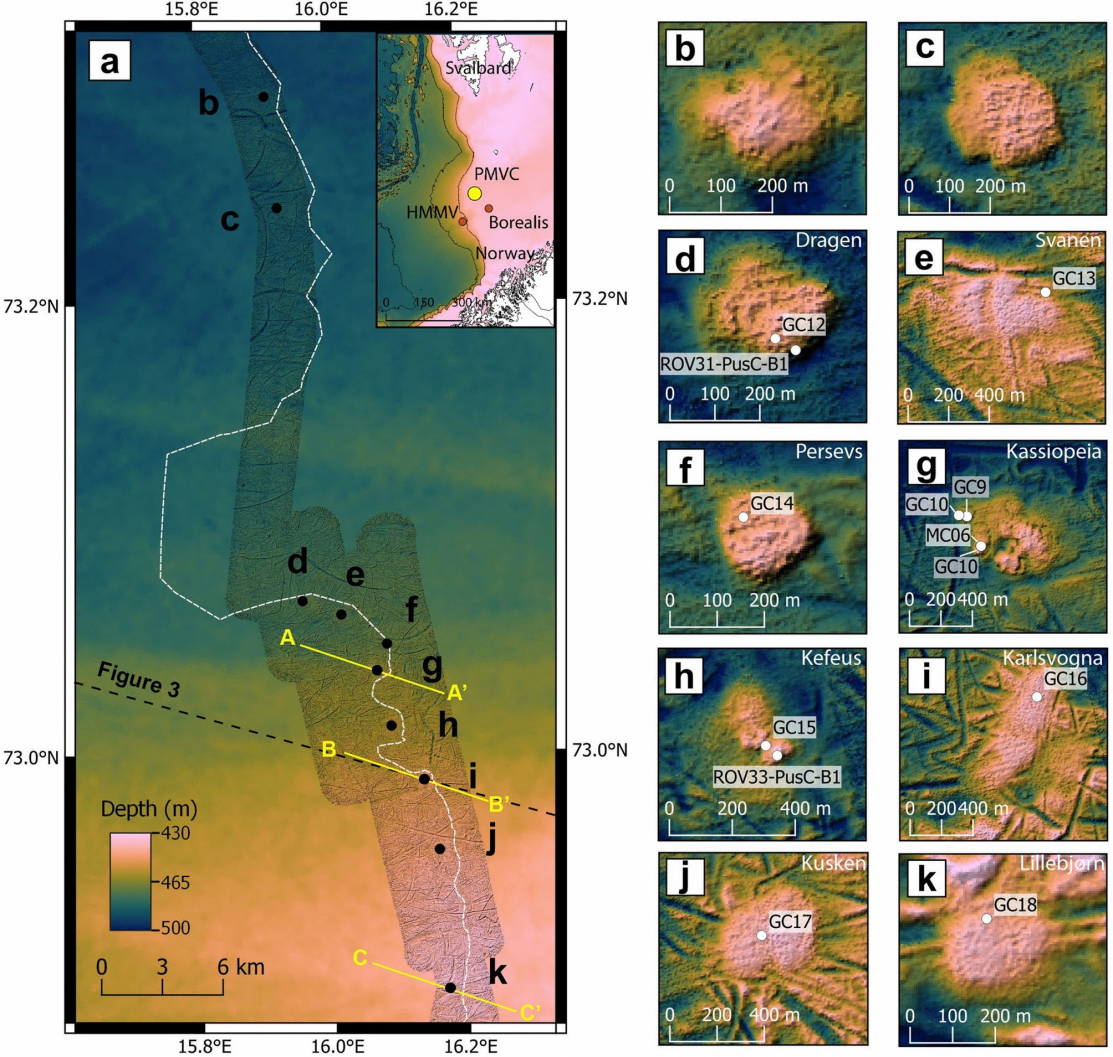
Overview of the study area showing the location and morphology of the Polaris Mud Volcano Complex (PMVC). (**a**) The inset map shows the geographic location of PMVC and the other two MVs in the SW Barents Sea, i.e. HMMV and Borealis. The area surveyed with high-resolution bathymetry is shaded, with IBCAO bathymetry in the background^45^. The ten mounds are indicated with black dots and are associated with letters from (**b**) to (**k**), referring to close-up views reported on the right. (**b**, **c**) have not been ground-truthed and are still unnamed. The color palette used in (**b**–**k**) has been adapted to emphasize the topography and does not match the depth scale in (**a**). The seafloor projection of the buried Pleistocene mega-slide head scar is shown with a dashed white line passing close to the mounds. The profile of the 2D seismic section of Fig. 3 is indicated with a NW–SE oriented dashed black line, whereas three shorter seismic profiles (AA′, BB′ and CC′) of Fig. 4 are shown in yellow. Color scale is accessible to people with color vision deficiencies^46^.

Additionally, in the caption of Figure 2,

“The freshly emitted material is flowing over older deposits colonized by sessile fauna (i.e. sea star in the picture).”

now reads:

“The freshly emitted material is flowing over older deposits colonized by sessile and mobile fauna (i.e. sea star in the picture).”

The original Article has been corrected.

