# Supplementary material for: A constellation of mud volcanoes originated from a buried Arctic mega-slide, Southwestern Barents Sea
Source: Sci Rep. 2025 Apr 30;15:15161. doi: 10.1038/s41598-025-99578-5 (PMC12043814; doi:10.1038/s41598-025-99578-5)
Supplement: Supplementary file 1 — Supplementary Information. [file 41598_2025_99578_MOESM1_ESM.docx]

Supplementary Information to:

A constellation of mud volcanoes originated from a buried Arctic mega-slide, Southwestern Barents Sea

Claudio Argentino,^1^ Rune Mattingsdal,^2^ Tor Eidvin^3^, Sverre Ekrene Ohm^4^, Giuliana Panieri^1,5^

^1^ Department of Geosciences, UiT The Arctic University of Norway, NO-9037 Tromsø, Norway

^2^ Norwegian Offshore Directorate, NO-9406 Harstad, Norway.

^3^ Retired from the Norwegian Offshore Directorate, NO-4003 Stavanger, Norway.

^4^ Department of Energy Resources, University of Stavanger, 4021 Stavanger, Norway

^5^ Institute of Polar Sciences, National Research Council (CNR-ISP), 30172 Venice Mestre, Italy

* Corresponding Author: Claudio Argentino claudio.argentino@uit.no

**Text:** Biostratigraphic analyses and sedimentology of mud volcano samples

**Supplementary Table S1:** Seafloor features observed during the ROV dives

**Supplementary Figure S1:** Multibeam-derived backscatter map of the Polaris mud volcanoes.

**Supplementary Figure S2:** n-Alkanes chromatograms

**Supplementary Figure S3:** Hopanes chromatograms and maturity proxies

**Supplementary Figure S4:** Steranes chromatograms and paleo-depositional proxies

**Supplementary Figure S5:** Pristane (Pr)/n-C17 vs Phytane (Ph)/n-C18 diagram

**Biostratigraphic analyses and sedimentology of mud volcano samples.**

The foraminiferal assemblages in the sediment samples collected from the mud volcanoes Kassiopeia (GC9, GC10, GC11, MC-06_006), Dragen (GC12), Persevs (GC14), Kefeus (GC15), Karlsvogna (ROV33-PusC-B1), Kusken (GC17) and Lillebjørn (GC18) are similar. In contrast, the samples from Svanen (GC13) and Dragen (ROV31-PusC-B1) display a markedly reduced species diversity compared to the others. Planktonic foraminiferal assemblages have been correlated with the zonation of Spiegler and Jansen^1^ for the Neogene on the Vøring Plateau. These assemblages are dominated by the sinistral (sx) cold-water dwelling species *Neogloboquadrina* *pachyderma* and both encrusted and not-encrusted varieties are present. Warm water dwelling planktonic foraminifera like dextral (dx) *N.* *pachyderma* and *Globigerina* *bulloides* are less frequent and rare in most samples. However, in Kassiopeia (GC10), Dragen (GC12) and Persevs (G14), *N. pachyderma* (dx) is common. In the Norwegian Sea, the encrusted variety of *N. pachyderma* (sx) occurs frequently from 1.8 Ma but is sporadic in older sediments^1^.

Benthic foraminifera align with the micropaleontological Cenozoic zonation of King^2^ for the North Sea. Nearly all the forms are extant species typically associated with Pleistocene to recent deposits on the Norwegian Shelf, confirming remobilization of ancient sediment. The assemblages consist mainly of cold-water forms including *Nonion* *labradoricum*, *Islandiella* *islandica*, *I.norcrossi*, *I. helenae*, *Elphidium* *excavatum*, *E.* *subarcticum*, *Haynesia orbiculare,* *Cassidulina* *reniforme* and *Virgulina* *loeblichi.* These benthic foraminiferal assemblages correlate with Subzone NSB 16x of King^2^ of Calabrian age (late early to late Pleistocene). Mixed within these Pleistocene assemblages we observed occasional to common occurrences of warm-water planktonic species such as *N. pachyderma* (dx) and *G. bulloides* typical for Holocene and recent sediments in the Norwegian Sea and shelf^3,4^. Likewise, it is noted rare occurrences of Holocene and recent benthic foraminifera typical on the Norwegian continental shelf including *Uvigerina peregrina* and *Angulogerina angulosa*^4–9^.

The benthic foraminiferal assemblage in Svanen (GC13) consists mainly of cold-water forms including *E.* *subarcticum, E.* *excavatum, C.* *reniforme, Nonion* *labradoricum, I. helenae, Haynesia orbiculare and I.* *islandica.* The sparce planktonic foraminiferal assemblage is dominated by the encrusted variety of *N*. *pachyderma* (sx), suggesting that most sediment originates from Pleistocene deposits younger than 1.8 Ma. Among the planktonic foraminifera, the few recorded right turned *N. pachyderma* may indicate a minor mixing with Holocene or recent sediments. In Dragen (ROV33-PusC-B1), planktonic foraminifera are rare and include a few encrusted and not encrusted variety of *N. pachyderma* (sx) and a few *N. pachyderma* (dx). Among benthic foraminifera, most species are rare, except *Nonion affinis* which is abundant. *Pullenia bulloides, Cibicides lobatulus* and the cold-water forms *N. labradoricum, E. excavatum,* *E.* *subarcticum* and *H.* *orbiculare* are common. A few specimens typical in the Holocene like *Uvigerina peregrina* and *Angulogerina angulosa* are also recorded. Jansen et al.^4^ described common *P. bulloides* and *N. affine* in the Holocene and the latest Weichselian sediments from the upper continental slope in the Møre Basin (Norwegian Sea). The sample from Dragen is a mixture of similar parts of Holocene and Pleistocene sediments.

Preliminary sedimentological observations of samples from the pushcores and core catchers from the different mud volcanoes indicate a heterogeneous composition including sparse pebbles (Wentworth scale 4-64 mm) and cobbles (Wentworth scale 64-256 mm). The sand fractions are dominantly quartzose, but some grains consist of sedimentary rocks, and a few consist of coal. The granules, pebbles and cobbles consist mainly of sedimentary rocks, but some consist of chalk, some are mollusk fragments and a few consist of coal.

**Supplementary Table S1** Seafloor features observed during the ROV dives

| Mud volcano | Latitude (DD) | Longitude (DD) | Mud flow/pool | Seep habitats | MDAC |
| --- | --- | --- | --- | --- | --- |
| Dragen | 73.2912 | 15.9120 |  | X |  |
| Svanen | 73.2418 | 15.9293 | n.a. | n.a. | n.a. |
| Persevs | 73.0679 | 15.9581 |  | X | X |
| Kassiopeia | 73.0617 | 16.0196 | X | X | X |
| Kefeus | 73.0483 | 16.0866 | X | X | X |
| Karlsvogna | 73.0367 | 16.0662 |  | X | X |
| Kusken | 73.0117 | 16.0920 | n.a. | n.a. | n.a. |
| Lillebjørn | 72.9879 | 16.1402 | n.a. | n.a. | n.a. |

A comprehensive table compiling direct seafloor observations of mud flows/pools, seep habitats, and methane-derived carbonates, as preliminarily marked onboard. Future image analyses will provide a more complete overview of the distribution of these features. ROV dives were conducted on Dragen, Persevs, Kassiopeia, Kefeus, Karlsvogna; ROV dive on Kusken was aborted due to technical issues. No ROV dives on Svanen, Kusken and Lillebjørn due to time restrictions. MDAC = methane-derived authigenic carbonates


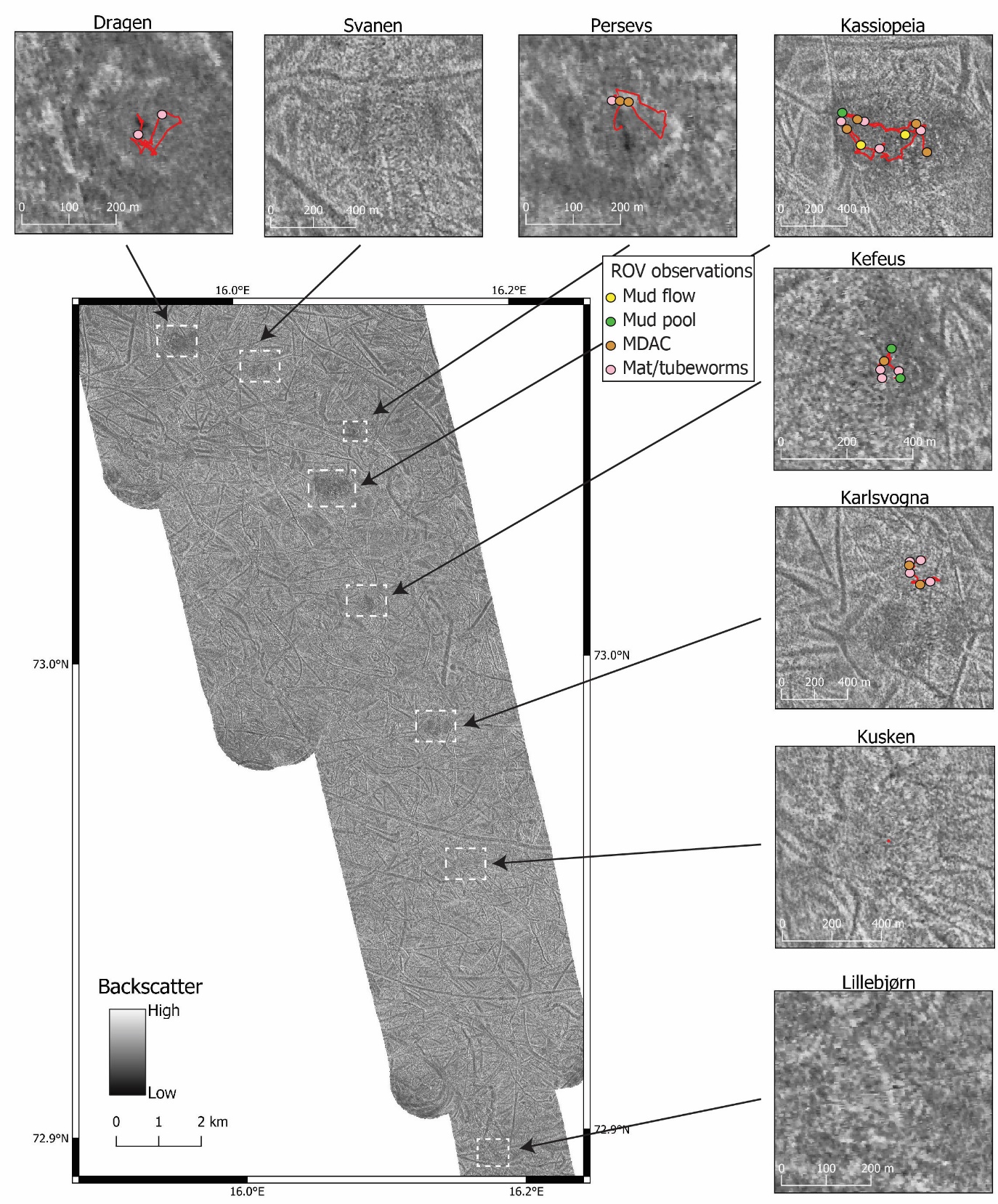


**Supplementary Figure S1.** Multibeam-derived backscatter map of the Polaris mud volcanoes. Overall, the mud volcanoes show low backscatter compared to the background seafloor, which indicates soft and uncompacted muddy lithology. Very low intensity is found in correspondence of mud flows and circular features (gryphons/pools). Bright spots correspond to seep carbonate outcrops (MDAC). The ploughmarks appear as paired high-backscatter lineations separated by a low backscatter central stripe, corresponding to coarser and finer-grained material respectively.


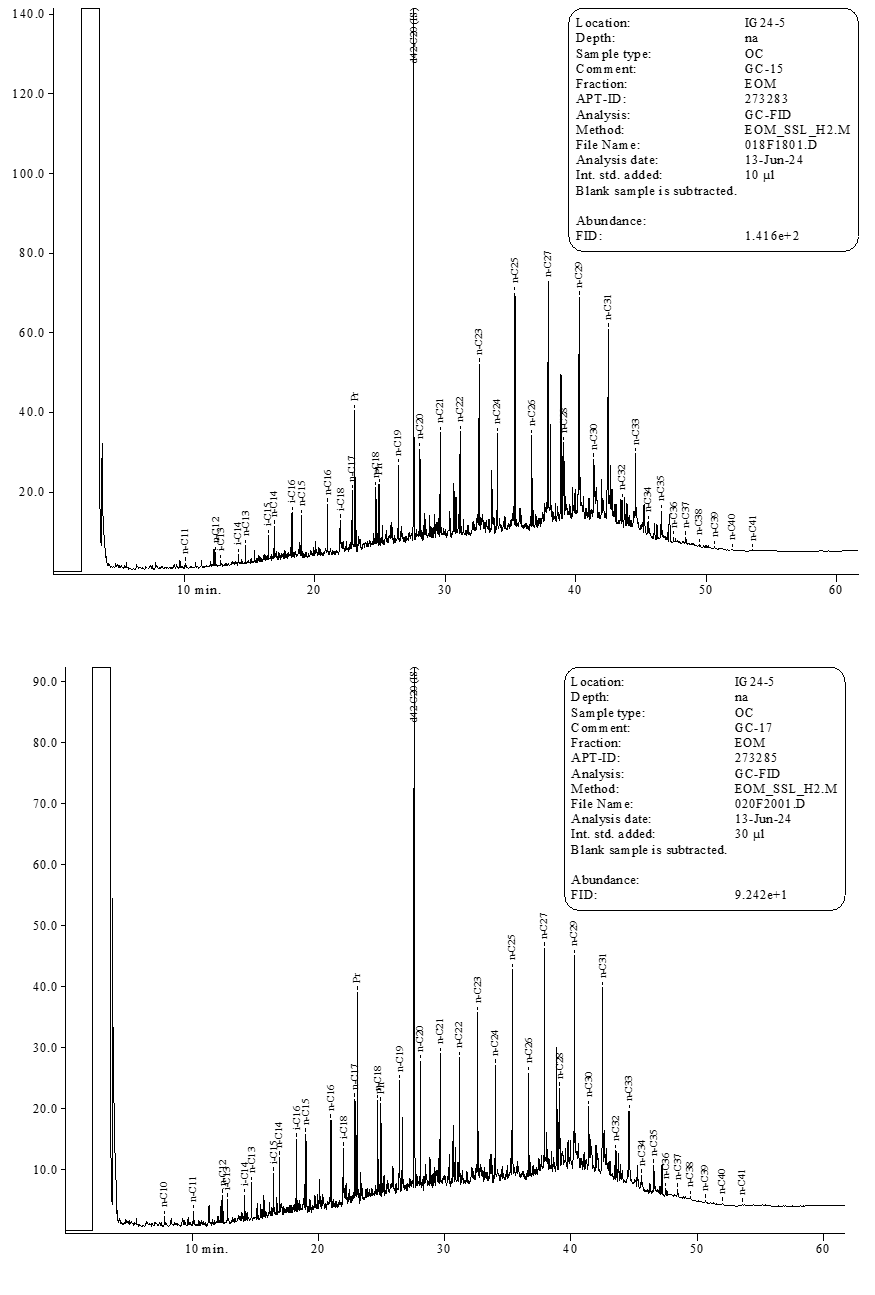


UCM

UCM

**Supplementary Figure S2**: Extract gas chromatograms showing n-alkanes distribution in sediment samples from gravity cores GC-15 and GC-17, collected from Kefeus and Kusken mud volcanoes, respectively. Both gas chromatograms displays and odd/even n-alkane dominance in the n-C_23_ to n-C_33_ range and the presence of unresolved complex mixture (UCM). The relatively high concentration of iso-alkanes relative to n-alkanes in the front end of the chromatograms and the UCM indicate biodegradation.


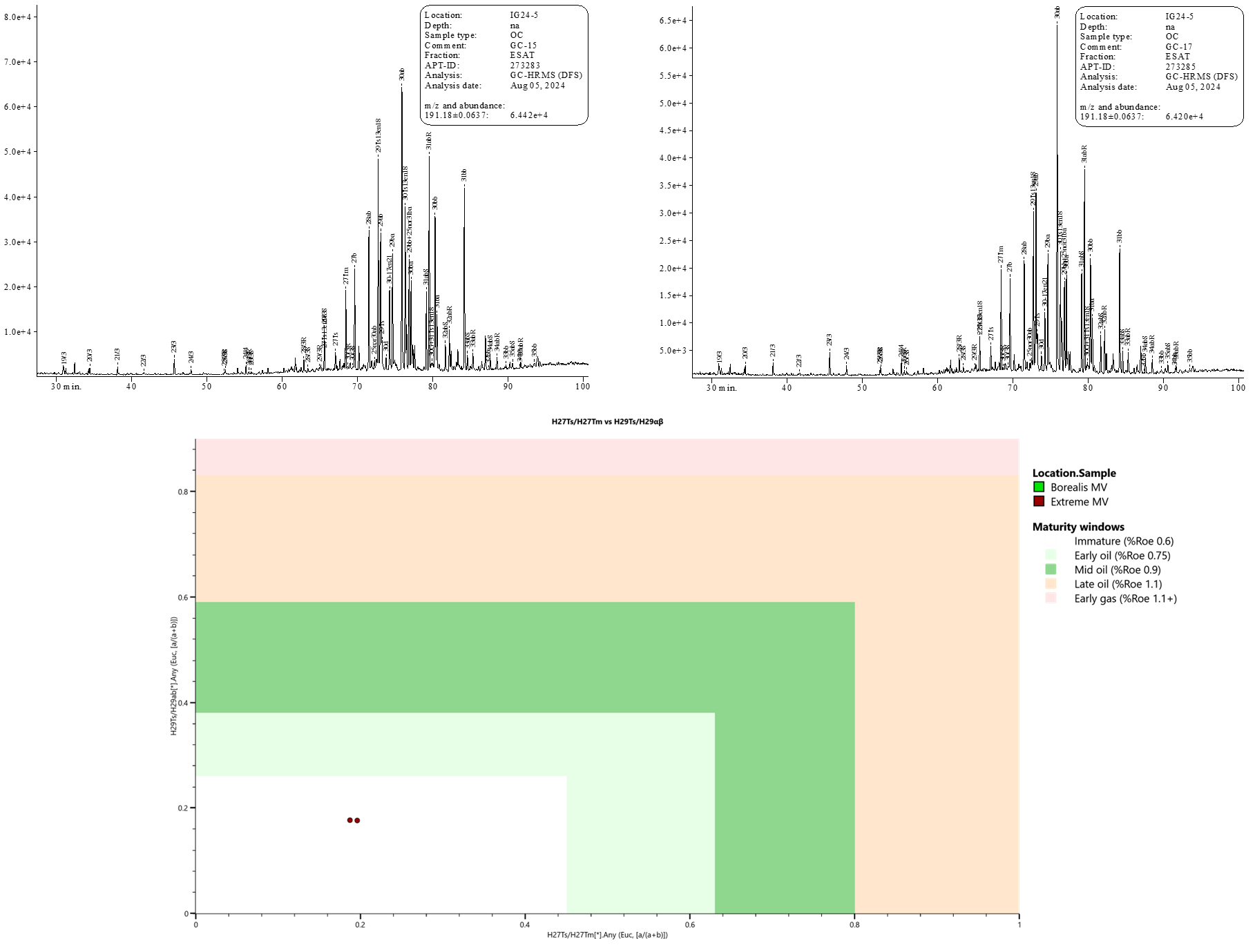


Early gas (%Roe 1.1+)

Mid oil (%Roe 0.9)

Late oil (%Roe 1.1)

Early oil (%Roe 0.75)

Immature (%Roe 0.6)

H29Ts/H29ab

1.0

0.8

0.6

0.4

0.2

0.0

0.0

0.2

0.4

0.6

0.8

H27Ts/H27Tm

**Supplementary Figure S3**: Hopanes distribution in the m/z 191 fragmentograms (top figures) and extrapolated hopane ratios H29Ts/H29ab vs H27Ts/H27Tm areused for assessing the degree of matury of organic matter sourcing the extracts^10^ (bottom figure). Hopanes derives from the degradation of hopanoids, which are biomarkers found in bacteria, algae, and higher plants. Structural changes occurring during the thermal maturation of organic matter can be quantified through specific hopane ratios^11,12^. Overall, hopanes in the Polaris samples suggest an immature source for the oil.


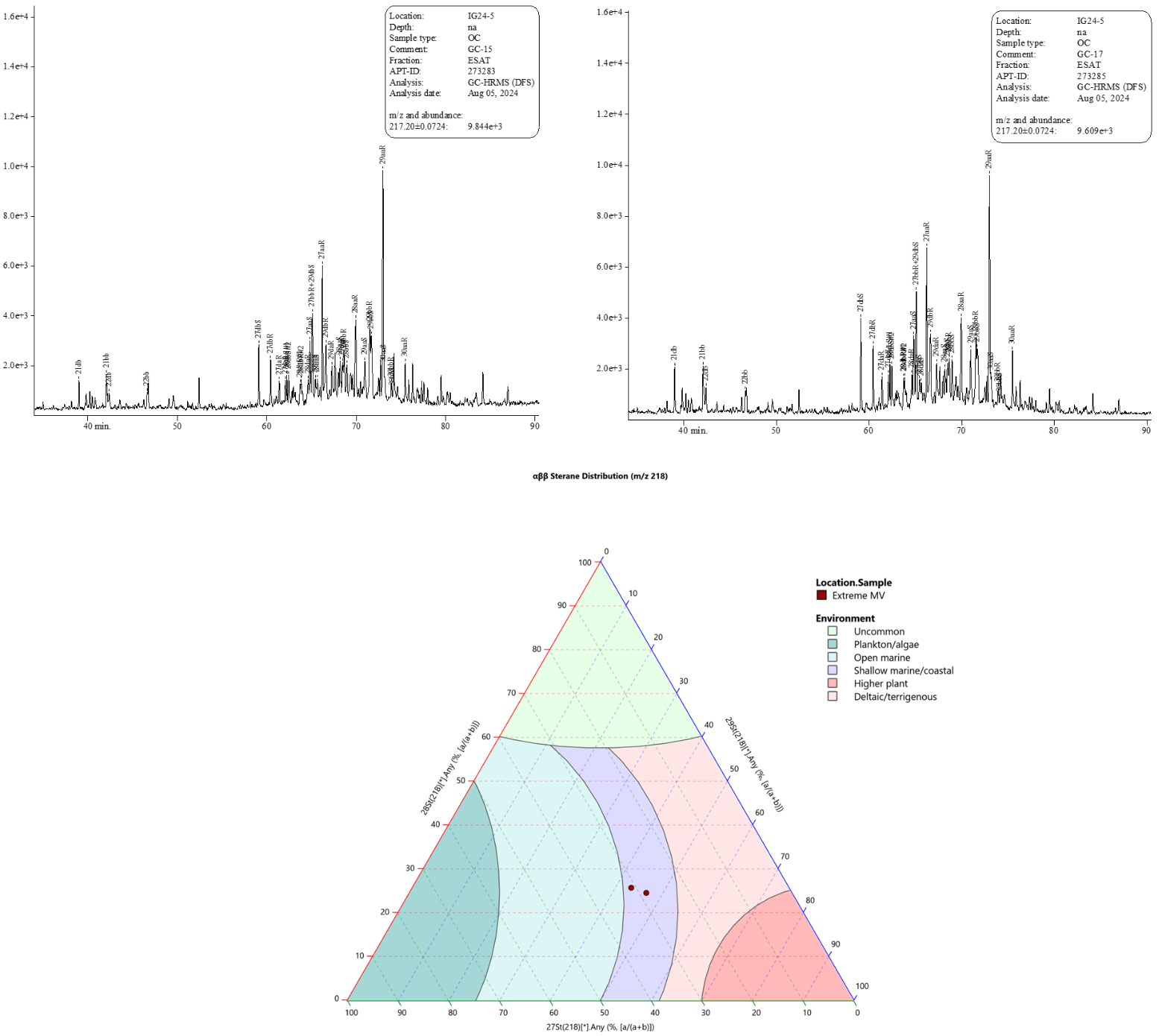


%C_29_

%C_27_

%C_28_

αββ Sterane distribution (m/z 218)

**Supplementary Figure S4**: Sterane distribution in the m/z 217 fragmentograms (top figures) and extrapolated sterane ternary diagram of the percentage of C_27_, C_28_, and C_29_ steranes obtained from the m/z 218 fragmentograms (bottom diagram). Steranes are derived from steroids contained in eukaryotic organisms such as algae and higher plants. Steranes can be used for interpreting the paleo-depositional environments (modified after Huang and Meinschein^13^) (bottom figure). The steranes in Polaris samples indicate a shallow marine/coastal environment source for the oil.


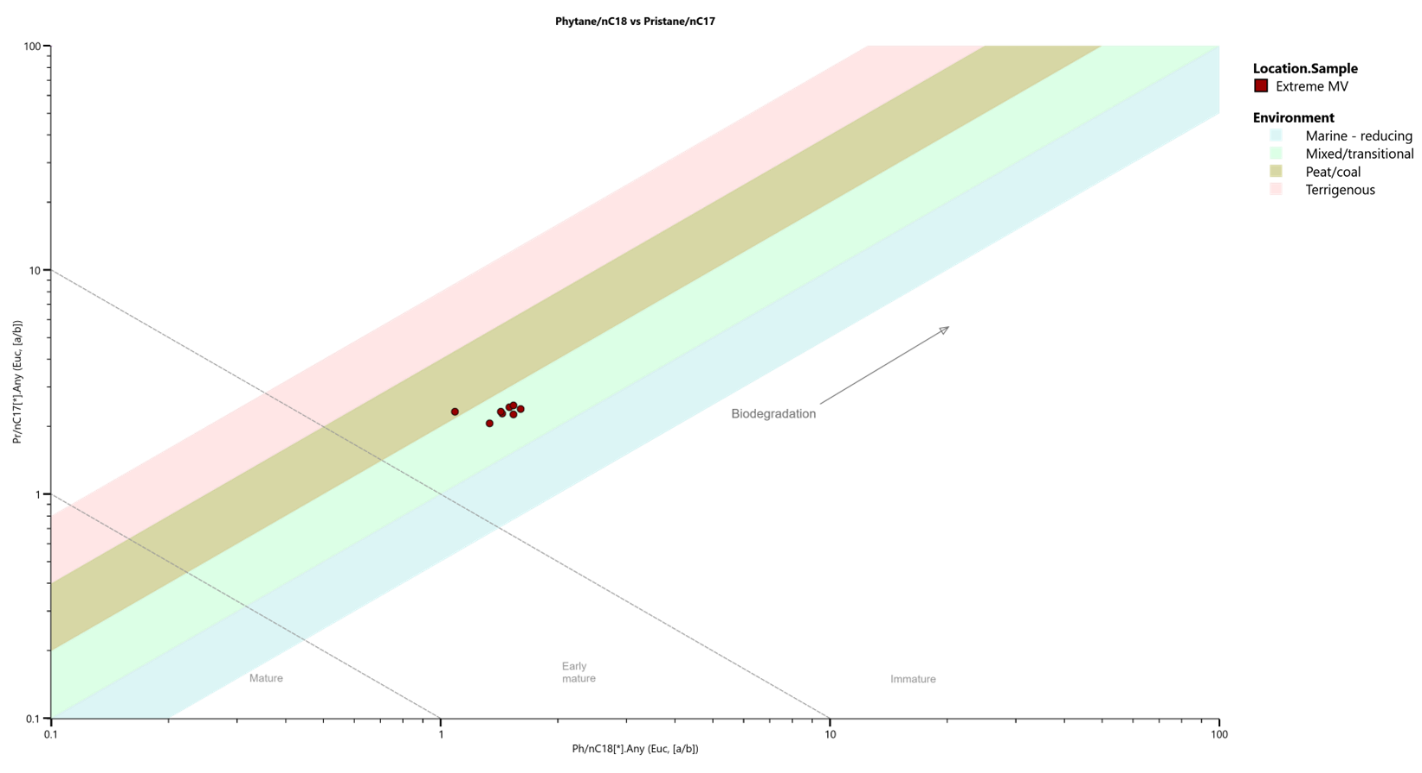


Mature

Immature

Early mature

Biodegradation

Terrigenous

Peat-coal

Mixed-transitional

Marine-reducing

Pr/n-C_17_

0.1

1

10

100

100

10

1

Ph/n-C_18_

0.1

**Supplementary Figure S5**: Pristane (Pr)/n-C_17_ vs Phytane (Ph)/n-C_18_ diagram used for assessing the depositional environment, redox conditions, and source of organic matter (modified after Lijmbach^14^). Polaris samples fall in the fields of immature, mixed/transitional marine environment and suggest ongoing biodegradation.

**References**

1. Spiegler, D. & Jansen, E. Planktonic foraminifer biostratigraphy of Norwegian Sea sediments: ODP Leg 104. in vol. 104 682–696 (1989).

2. King, C. Cenozoic of the North Sea. in *Stratigraphical Atlas of Fossil Foraminifera* 418–489 (Ellis Horwood Ltd., Chichester, 1989).

3. *Proceedings of the Ocean Drilling Program, 104 Scientific Results*. vol. 104 (Ocean Drilling Program, 1989).

4. Jansen, E. *et al.* Late Weichselian paleoceanography of the southeastern Norwegian Sea. in *Norsk Geologisk Tidsskrift* vol. 63 117–147 (1983).

5. Klitgaard Kristensen, D. & Sejrup, H. P. Modern benthic foraminiferal biofacies across the Northern North Sea. *Sarsia* **81**, 97–106 (1996).

6. Sejrup, H.-P. *et al.* Benthonic foraminifera in surface samples from the Norwegian continental margin between 62 degrees N and 65 degrees N. *The Journal of Foraminiferal Research* **11**, 277–295 (1981).

7. Hald, M. & Vorren, T. O. Modern and Holocene foraminifera and sediments on the continental shelf off Troms, North Norway. *Boreas* **13**, 133–154 (1984).

8. Hald, M. & Steinsund, P. I. Distribution of surface sediment benthic Foraminifera in the southwestern Barents Sea. *The Journal of Foraminiferal Research* **22**, 347–362 (1992).

9. Mackensen, A., Sejrup, H. P. & Jansen, E. The distribution of living benthic foraminifera on the continental slope and rise off southwest Norway. *Marine Micropaleontology* **9**, 275–306 (1985).

10. Lerch, B., Karlsen, D. A., Matapour, Z., Seland, R. & Backer-Owe, K. Organic geochemistry of barents sea petroleum: Thermal maturity and alteration and mixing processes in oils and condensates. *Journal of Petroleum Geology* **39**, 125–148 (2016).

11. J. Michael Moldowan (2), Wolfgang K. Relationship Between Petroleum Composition and Depositional Environment of Petroleum Source Rocks. *Bulletin* **69**, (1985).

12. Dembick-Jr., H. *­ Petroleum ­ Geochemistry for Exploration and Production*. (2017).

13. Huang, W.-Y. & Meinschein, W. G. Sterols as ecological indicators. *Geochimica et Cosmochimica Acta* **43**, 739–745 (1979).

14. Lijmbach, W. On the origin of petroleum. in *Proceedings of the 9th World Petroleum Congress* (Applied Science Publishers, London, 1975).
